# Immune pathways and defence mechanisms in honey bees *Apis mellifera*

**DOI:** 10.1111/j.1365-2583.2006.00682.x

**Published:** 2006-10-01

**Authors:** J D Evans, K Aronstein, Y P Chen, C Hetru, J-L Imler, H Jiang, M Kanost, G J Thompson, Z Zou, D Hultmark

**Affiliations:** * USDA-ARS Bee Research Laboratory Beltsville, MD, USA; † USDA-ARS Beneficial Insects Laboratory Weslaco, TX, USA; ‡ Institut de Biologie Moléculaire et Cellulaire CNRS, Strasbourg, France; § Dept. Entomology, Oklahoma State University USA; ¶ Dept. Biochemistry, Kansas State University USA; ** School of Biological Sciences, University of Sydney NSW, Australia; †† Umea Centre for Molecular Pathogenesis, Umea University Sweden

**Keywords:** innate immunity, comparative genomics, antimicrobial peptide, American foulbrood

## Abstract

Social insects are able to mount both group-level and individual defences against pathogens. Here we focus on individual defences, by presenting a genome-wide analysis of immunity in a social insect, the honey bee *Apis mellifera*. We present honey bee models for each of four signalling pathways associated with immunity, identifying plausible orthologues for nearly all predicted pathway members. When compared to the sequenced *Drosophila* and *Anopheles* genomes, honey bees possess roughly one-third as many genes in 17 gene families implicated in insect immunity. We suggest that an implied reduction in immune flexibility in bees reflects either the strength of social barriers to disease, or a tendency for bees to be attacked by a limited set of highly coevolved pathogens.

## Introduction

While evident in social organisms ranging from humans to birds (Brown & Brown, 2004; Masuda *et al*., 2004), the impacts of sociality on disease are especially vivid within social insect colonies. Here, typically thousands of individuals interact in close quarters, at densities far exceeding those of even the most crowded vertebrate social groups (Wilson, 1971). This density, coupled with a relatively homeostatic nest environment and the presence of stored resources, makes social insects attractive targets for disease agents (Schmid-Hempel, 1998). As expected based on their parasite and pathogen pressures, social insects have evolved both individual and group strategies to combat disease. Grooming, nest hygiene and other behavioural traits found throughout the social insects can reduce the impacts of pathogenic bacteria, fungi and parasitic mites. For example, ‘hygienic behaviour’ first described for honey bees (Rothenbuhler, 1964) is now a classical example of a social defence, whereby workers identify and remove infected larvae from among the healthy brood (Spivak & Reuter, 2001). Other defences enabled by sociality include the construction of nests from antimicrobial materials (Christe *et al*., 2003), the raising of offspring in sterile nurseries (Burgett, 1997), social ‘fever’ in response to disease (Starks *et al*., 2000), transference of immune traits (Traniello *et al*., 2002; Sadd *et al*., 2005), and heightened risk-taking by infected individuals (Schmid-Hempel, 2005). Like most eukaryotes, colony members also possess individual defences, including immune responses toward disease agents (Casteels-Josson *et al*., 1994; Evans, 2004). The recent sequencing of the honey bee genome (Honey Bee Genome Sequencing Consortium, 2006) allows the first global analysis of immune components in honey bees, and the second opportunity (after humans) to use genomic insights to better understand disease resistance in a highly social organism.

Insects have diverse mechanisms to combat infection by pathogens. Many insects are protected by a layer of antimicrobial secretions on their exterior, and by a gut environment that is hostile to pathogens. When pathogens move beyond these defences, the epithelium is often sufficient to stop further progress. Should pathogens defeat the morphological defences of insects, they are often met by efficient cellular and humoral immune defences. Insect immunity shows many parallels to the innate immune responses of humans and other vertebrates, involving a diverse set of actions including the secretion of antimicrobial peptides, phagocytosis, melanization and the enzymatic degradation of pathogens (Hoffmann, 2003; Hultmark, 2003). Further, insect immune pathways share both an overall architecture and specific orthologous components with the innate immune system of vertebrates (Beutler, 2004). This suggests both a shared root for these immune pathways and selection to conserve many components over hundreds of millions of years.

In the first part of this paper, we propose honey bee models for four non-autonomous pathways implicated in inducible host defence, Toll, Imd, Janus kinase (JAK)/STAT and JNK (Boutros *et al*., 2002), based primarily on extensive searches for orthologues to well-studied fruit fly, mosquito and moth species. While these pathways engage in cross-talk and can direct some of the same immune effectors, they have well-defined structures and interaction sets, and are best tackled as individual entities. Most honey bee components for these pathways remain to be validated by functional tests, yet we feel that the presented models serve two important purposes. First, they point toward the most likely orthologues involved with all stages of the immune response, thus setting the stage for postgenomic functional work on honey bee immunity. Second, the models themselves show intriguing differences between species for these canonical immune pathways with respect to gene losses and duplications.

Next, we show that many immune-gene families in bees appear to be reduced in number, when compared to *Drosophila* and *Anopheles*. While genome-wide analyses of the honey bee have identified many gene families with reduced diversity (Honey Bee Genome Sequencing Consortium, 2006), such reductions appear to be especially pervasive in the immune system. These reductions hold for each stage of immunity, from recognition and signalling to immune effectors. We couple gene-family data with data on specific orthologues to test five hypotheses: (1) missing genes are not represented in the current draft honey bee genome assembly but are present in the genome; (2) immune-related genes in the bee have diverged especially quickly at the sequence level and, as such, have escaped annotation based on sequence similarity to other species; (3) honey bees enact immune responses using pathways and/or components not currently identified as immune players in other insects; (4) honey bees are targeted by a small set of coevolved pathogens and their immune systems are thereby tuned to these pathogens at the expense of being responsive to a wider range of threats; and (5) ‘social’ defences and barriers in honey bee colonies are effective in reducing pathogen pressure and, as such, bees are not as reliant as other insects on individual immune responses.

## Results

### Overview of immune pathways

**Figure 1:**
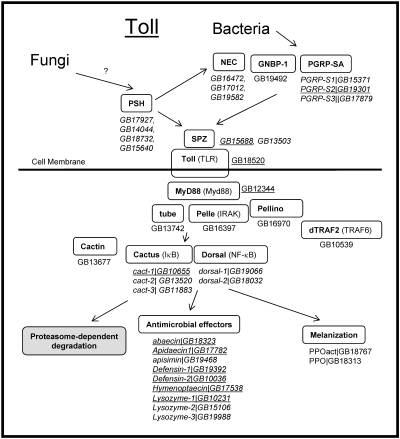
Candidate honey bee members for the Toll pathway. Names are given for the *Drosophila* pathway components, along with vertebrate orthologues (in parentheses). Honey bee matches given as named during the genome project. Honey bee names in italics refer to genes with close paralogues which cannot readily be distinguished with respect to pathway components from *Drosophila*. Underlining indicates genes shown to be transcriptionally up-regulated after immune challenge.

**Figure 2:**
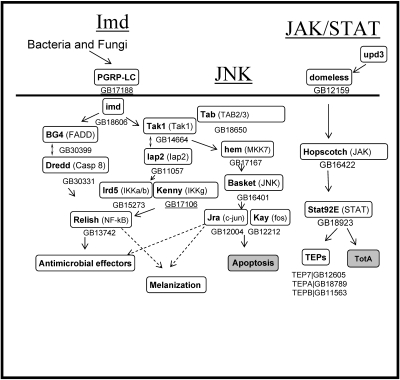
Candidate honey bee members for the Imd, JNK and JAK/STAT pathways, below names for *Drosophila* pathway components along with vertebrate orthologues (in parentheses). Honey bee matches presented as named during the genome project. Honey bee names in italics refer to genes with close paralogues which cannot readily be distinguished with respect to pathway components from *Drosophila*. Underlining indicates genes shown to be transcriptionally up-regulated after immune challenge.

Honey bees possess apparent orthologues for the core members of each of the four pathways implicated in immunity (Figs 1 and 2, Supplementary Material Table S1) and precise 1 : 1 : 1 orthology between honey bees and the flies *Drosophila melanogaster* and *Anopheles gambiae* is evident for most pathway members, especially for the intracellular components. Of the dozens of described actors in four signalling pathways predicted to play a role in insect immunity, only one protein appears to be completely absent in the bee genome: the ligand *unpaired* from the JAK/STAT pathway. The presence of the JAK/STAT cytokine receptor *domeless* and all other members of this pathway (Fig. 2) suggest that JAK/STAT remains functional in honey bees and is triggered by a currently unrecognized ligand.

### Toll pathway

**Figure 3:**
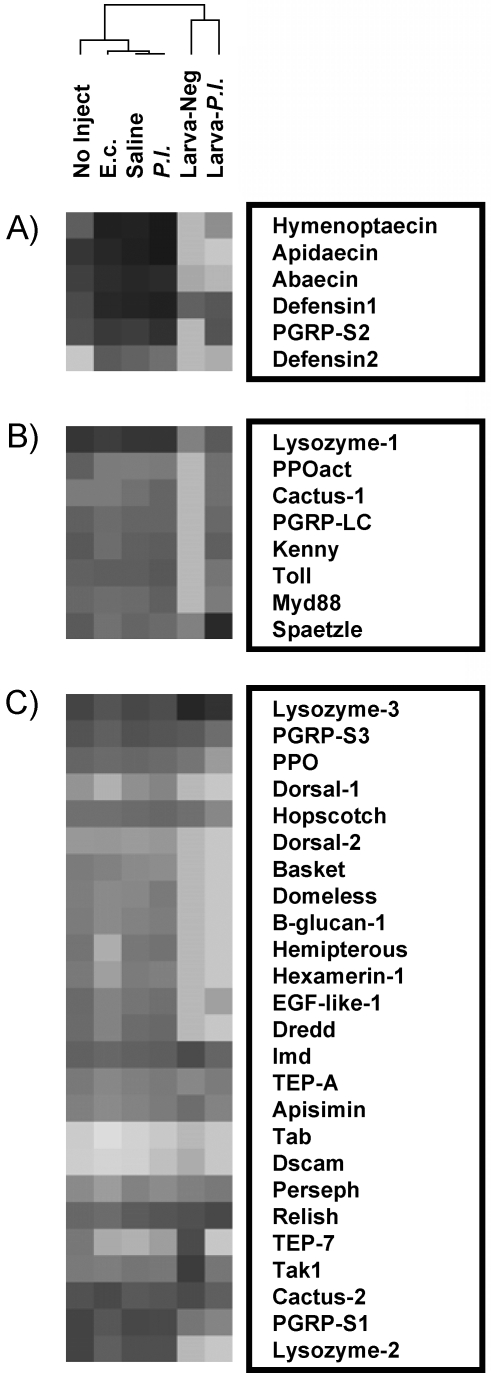
Transcript abundances for immune candidate genes in adult workers 24 h after injections of *Escherichia coli* (Ec), saline buffer, or the bee pathogen *Paenibacillus larvae*, and controls (left four columns). Two columns on right show transcript abundances in 2nd-instar larvae challenged orally with an infective dose of *P. larvae* or unchallenged controls. Cluster A = genes strongly up-regulated by adult injection or wounding, Cluster B = genes up-regulated in infected larvae, Cluster C = genes down-regulated or minimally changed in challenged bees.

Insect Toll and the Toll-like receptors (TLRs) are transmembrane signal transducing proteins that play critical roles in both immunity and development. They are orthologous to mammalian TLRs, all of which have been implicated in immunity (Beutler, 2004). In *Drosophila*, the Toll signalling pathway is enacted when the cytokine-like molecule Spaetzle binds to the extracellular domain of the transmembrane receptor Toll. The *Drosophila* genome encodes a family of six Spaetzle-related molecules, that are believed to function as ligands for the nine *Drosophila* Toll receptors (Parker *et al*., 2001). Two plausible Spaetzle orthologues are evident in the bee genome (GB15688 and GB13503; Fig. 1), and functional tests will be needed to determine which act as Toll-binding cytokines. Following conformational changes of the activated receptor, several intracellular death-domain (DD) containing proteins are recruited to form a receptor complex. Activation of this complex leads to the degradation of the NF kappa B inhibitor (IκB) Cactus and subsequent nuclear translocation of the NF-κB transcription factor Dorsal (or the Dorsal-related immune factor, Dif, in *Drosophila* (Royet *et al*., 2005). Two homologues of Dorsal were found in the honey bee genome (Fig. 1), neither of which was orthologous with Dif. This lends support to the view that Dif is a highly derived branch found in brachyceran flies but absent from other insects. In mosquitoes (Shin *et al*., 2005), and arguably honey bees, Dorsal (called REL1 in mosquitoes) is a functional alternate for Dif. Functional tests can help determine which of the two dorsal paralogues is the key transcription factor for this pathway. The intracellular components Tollip, Pellino, Cactin and TNF receptor associated factor-2 (TRAF-2) are believed to aid the main players of this pathway, and all appear to be present in both fly species as well as the honey bee. Candidate effectors for the immune-related Toll pathways in honey bees include a compliment of antimicrobial peptides, the melanizing agent phenoloxidase and three lysozymes. While it has not been confirmed that these effectors are triggered by the Toll pathway as opposed to other pathways described below, it is evident that some of the bee effectors are responsive to pathogens and/or mechanical wounding of bees (Fig. 3).

### Imd pathway

While Toll signalling in flies serves a dual purpose in development and immunity, the signalling process activated by peptidoglycan recognition protein (PGRP)-LC and Imd is specific for antimicrobial defence and is dispensable for normal development (Hultmark, 2003). Via the NF-κB-like transcription factor Relish, this signalling induces transcription of all major antimicrobial effector peptides in *Drosophila*. In *Drosophila*, Imd signalling is often said to be specific for Gram-negative bacteria, although Gram-positive bacteria with diaminopimelic acid-type peptidoglycans are at least as strong as elicitors. A weaker response is also seen to other types of peptidoglycan and even to fungi (Hultmark, 2003; Werner *et al*., 2003; Stenbak *et al*., 2004). This broad specificity is caused by the three alternative splice forms of *Drosophila* PGRP-LC, which carry different peptidoglycan recognition domains (Werner *et al*., 2003; Mellroth *et al*., 2005. Chang *et al*., 2006). Interestingly, the Imd signalling pathway is highly conserved in the honey bee, with plausible orthologues for all components (Fig. 2). While this strongly suggests that Imd signalling is similar in flies and bees, it does not necessary imply similar biological roles.

Besides the activation of Relish, Imd signalling also leads to activation of components of the JNK signalling pathway (Boutros *et al*., 2002), and recent evidence indicates that this pathway can provide both positive and negative feedback for the expression of the antimicrobial peptides (Wojda *et al*., 2004). Plausible orthologues for each of the major components of the JNK signalling were also identified in the honey bee genome (Fig. 2).

### JAK/STAT pathway

The JAK/STAT signalling pathway may also contribute to innate immunity by induction of complement-like factors and the overproliferation of haemocytes. JAK/STAT appears to be initiated via cytokine-like molecules in blood cells (Agaisse & Perrimon, 2004). In flies, the extracellular glycosylated protein Upd acts as a ligand that activates the JAK/STAT pathway, which in turn promotes phagocytic activity of haemocytes. The JAK/STAT pathway has also recently been shown to participate in an antiviral response in *Drosophila* (Dostert *et al*., 2005). Honey bee homologues for the *Drosophila* JAK/STAT signalling pathway (Fig. 2) comprise the cytokine receptor domeless (Dom), JAK tyrosine kinase (Hopscotch), the STAT92E transcription factor and two negative pathway regulators SOCS (suppressor of cytokine signalling) and PIAS (protein inhibitor of activated STAT). Orthologues of two recently identified components of this pathway (Baeg *et al*., 2005; Muller *et al*., 2005), the tyrosine phosphatase Ptp61F (XP392429) and the WD40- and bromo-domain-containing protein BRWD3 (XP395263), are also present in the honey bee. Although the key ligand (Upd) for the JAK/STAT pathway was not found in the honey bee genome, the presence of the gp130 cytokine receptor homologue Domeless and all other members of the signalling pathway indicates that this mechanism may be common across insects and is intact in honey bees as well as in flies. In addition to up-regulating the complement-like thiolester-containing proteins (TEPs; Lagueux *et al.*, 2000; Boutros *et al*., 2002), the JAK/STAT pathway in *Drosophila* regulates expression of the Turandot (Tot) genes that encode humoral factors induced by severe stress (Ekengren & Hultmark, 2001; Ekengren *et al*., 2001; Agaisse *et al*., 2003). None of the Tot factors (Tot A-Z) are apparent in honey bees.

### Overall gene family diversity

While honey bees appear to have maintained each of the known insect immune-related pathways, they appear to do so with a reduced number of paralogous members. When comparing a set of 17 gene families and functional groups implicated in immune responsiveness (Christophides *et al*., 2002), honey bees have substantially lower paralogue counts than either *Drosophila* or *Anopheles* (Table 1). The 71 genes placed into these groups for honey bees are in sharp contrast to the 196 and 209 found in *Drosophila* or *Anopheles*, respectively. Bees have the lowest gene counts for 12 of the 17 families and are tied for the lowest count two more times. *Drosophila* and *Anopheles* were lowest for only one family each (defensins and dorsal, respectively). In contrast, *Drosophila* and *Anopheles* show the highest paralogue counts for this triad seven and eight times, respectively, versus once in bees (for the Toll-pathway candidate cactus, with three copies). These rankings are significantly different under an ordinal contingency-table analysis (*P* < 1.0 × 10^−4^), and reflect differences for genes involved with pathogen recognition and signalling, as well as effectors.

**Table 1:** Gene counts for a subset of gene families implicated in insect immunity. *Anopheles gambiae* and *Drosophila melanogaster* counts based on Christophides *et al*. (2002,^ 2004^) and newer analyses

Gene family	*A. mellifera*	*A. gambiae*	*D. melanogaster*
Recognition			
PGRP-S	3	3	7
PGRP-L	1	4	6
B-glucan	2	6	3
Galectins	2	8	5
C-type lectins	10	22	35
Fibrinogen-domain	2	57	13
Signalling			
CLIP serine proteases	18	41	37
Serpin*	7	14	28
Toll	5	11	9
Cactus	3	1	1
Dorsal	2	1	2
Relish	2	2	3
Effectors			
Prophenoloxidase	1	9	3
Defensins	2	4	1
Other immune peptides	4	5	19
Lysozyme	3	6	14
TEP	4	15	6
Total	71	209	196

* Includes two genes with serpin-like sequences.

### Pathogen recognition gene diversity

**Figure 4:**
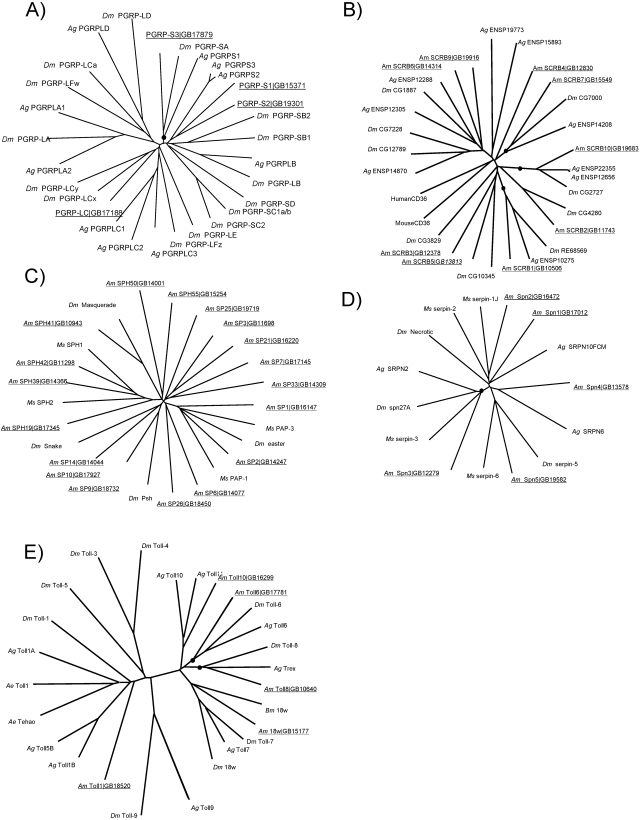
(A) Phylogenetic relationships estimated for peptidoglycan recognition protein (PGRP) family members for honey bees (underlined), *Drosophila melanogaster* (Dm), and *Anopheles gambiae* (Ag). Circles indicate apparent three-way orthologues for these three species. (B) Relationships between members of the Class B scavenger receptors, including the above insect members as well as mouse and human CD36 proteins. (C) Relationships between honey bee serine proteases (AmelSP) and serine-protease homologues (AmelSPH) and a representative subset of those from *Drosophila melanogaster* and *Manduca sexta* (Ms). (D) Phylogenetic relationships of honey bee serpins (AmelSpn) compared to homologues in Dm, Ag and Ms, colours as above. (E) Toll and Toll-like receptors for honey bees, Dm, Ag, *Bombyx mori* (Bm) and *Aedes aegypti* (Ae). Relationships derived by neighbor joining as described in the text.

PGRPs, major players in pathogen recognition (Hultmark, 2003; Steiner, 2004; Royet *et al*., 2005) are less diverse in honey bees versus flies and other insects for which genomic data exist (e.g. the moth *Bombyx mori*). There are only four PGRPs in the honey bee genome, compared to 13 and seven in *Drosophila* and *Anopheles*, respectively (Fig. 4A, Table 1). Further, bees show no capacity for the splice variation that contributions to diversify peptidoglycan recognition specificity in flies and mosquitoes. Specifically, the single membrane-bound PGRP in bees (PGRP-LC, GB17188) is similar to fly and mosquito PGRP-LC but lacks the potential to insert alternative peptidoglycan recognition domains by alternative splicing, a factor in recognition breadth for flies. PGRP-LC and PGRP-S2 are both up-regulated in honey bees after disease challenge (as in flies), suggesting that the products of these genes indeed play a defensive role (Fig. 3). As in *Anopheles*, there is only one Class C Scavenger Receptor (SR-C) in the honey bee. This group has diversified into four members in *Drosophila*, three of which show selective signs suggestive of an immune role (Lazzaro, 2005). There are 10 Class B scavenger receptors in the bee, a number roughly similar to that in the fly and mosquito (Fig. 4B, Table 1). Several other recognition classes also seem to be reduced in honey bees, including β-glucan recognition proteins (βGRPs), galectins and fibrinogen-related proteins (Table 1). Of these, the fibrinogen-domain genes are especially striking, due to the absence in bees of high lineage-specific diversification found in mosquitoes and *Drosophila* [resulting in 57 and 13 domain-family members, respectively (Christophides *et al*., 2004)].

Along with PGRP-LC and SR-C (Stuart & Ezekowitz, 2005), two other receptors were recently found to participate in the phagocytosis of infectious non-self in *Drosophila*, DSCAM and Eater. DSCAM, long implicated in neuronal development, was shown to have a likely role in the binding of bacteria by *Drosophila* haemocytes (Watson *et al*., 2005). This gene, which has > 12 000 potential splice variants in honey bees thanks to three sets of highly interchangeable exons (Graveley *et al*., 2004), is a very interesting candidate for determining the extent to which bees might better tune their immune response to specific pathogens. Another recently identified protein involved in *Drosophila* cellular immunity is Eater, a phagocytic receptor characterized by several repeats of an EGF motif in its extracellular domain (Kocks *et al*., 2005). Many genes with EGF motifs are present in the bee genome (e.g. GB14654, Supplementary Material Table S1), as in flies, although their orthology with Eater is unclear. Bees are also likely to engage in the encapsulation of endoparasites and pathogens, and show typical integrins (Honey Bee Genome Sequencing Consortium, 2006) implicated in lamellocyte encapsulation (Irving *et al*., 2005). None of these cellular immunity components appear to be more diverse in bees than in fly species (Supplementary Material Table S1).

### Signalling gene diversity

In addition to their function in digestion of food, serine proteases (SPs) in insects participate in regulatory cascade pathways in embryonic development and in immune responses (Kanost & Clarke, 2005). Many haemolymph SPs and serine-protease homologues (SPHs) implicated in immunity contain one or more clip domains at their amino terminus, which may regulate or localize the immune responses stimulated by protease cascades (Jiang & Kanost, 2000). Among the 57 SP-related proteins in the honey bee genome, 12 SPs and six SPHs contain at least one clip domain, significantly fewer than in *Drosophila* (24 SPs and 13 SPHs) (Ross *et al*., 2003) or *Anopheles* (26 SPs and 15 SPHs), and smaller than the number of clip domain SPs identified to date (*n* = 14) from expression data in *Manduca sexta* (Jiang *et al*., 2005). Additional phylogenetic and functional relationships among insect SPs and SPHs are discussed in Zou *et al*. (2006).

Serine protease inhibitors from the serpin superfamily regulate protease cascades in mammals and in arthropods (Reichhart, 2005). In insect haemolymph, serpins inhibit activated proteases to maintain homeostasis and prevent unregulated activation of immune responses such as melanization or Toll-mediated antimicrobial protein synthesis (Kanost & Clarke, 2005). In the honey bee genome, there are seven annotated genes encoding five serpins and two proteins with serpin-like regions (GB10078 and GB15070). The number of serpin genes in the honey bee is much lower than in *Drosophila* (28) or *Anopheles* (14), mirroring the reduced size of the protease gene family in bees.

Nine Toll-related receptor genes are known from the *Drosophila* genome and 10 from *Anopheles* (Tauszig *et al*., 2000; Christophides *et al*., 2004). In *Drosophila*, Toll is the primary family member implicated in immune-related function (Lemaitre *et al*., 1996), although it appears that *Drosophila* Toll-5 and Toll-9 (the paralogue that is structurally most similar to mammalian TLRs), are also involved in immune-related signalling (Ooi *et al*., 2002; Bilak *et al*., 2003). We have identified only five Toll-related genes in the honey bee: Toll1, Toll2/18w, Toll6, Toll8/Trex/Tollo and Toll10. Additional Toll members in *Drosophila* (Toll-3, -4, -5, -7 and -9) apparently reflect gene duplication events in the fly lineage (Fig. 4) or, in the case of Toll-9, arguably a loss in the honey bee and lepidopteran lineages. For instance, the ancestral Toll appears to have diverged into two different groups, Toll-1 and Toll-5, in flies. In contrast, Toll-1 remains the only member of this clade in honey bees. Similarly, the Toll-7/2 clade is represented only by a single honey bee homologue Am18w (Aronstein & Saldivar, 2005). Presumably, the ancestral Toll 7/2 gene was duplicated in flies following their divergence 300 Mya from the lineage leading to honey bees. A protein named *Apis mellifera* toll7 (Kanzok *et al*., 2004), seems more likely to be an orthologue of Toll-10. The five Toll receptors present in the *A. mellifera* genome (Toll-1, -6, -2/7, -8, -10) are also present in the sequenced genomes of other insects that belong to the orders Diptera, Lepidoptera and Coleoptera, with few exceptions. For example, whereas orthologues of Toll-6, -7 and -8 are found in *D. melanogaster* and *A. gambiae* (Diptera), *Bombyx mori* (Lepidoptera), and *Tribolium castaneum* (Coleoptera), Toll-1 appears to be absent from the genome of the lepidopteran insect *B. mori*, whereas Toll-10 appears to have been lost in the fruit fly. This suggests that these five genes encode the basic set of Toll receptors that was present in the common ancestor of these insects. These five receptors are highly expressed, in a dynamic and tissue-specific manner, during *Drosophila* embryogenesis (Kambris *et al*., 2002). Of note, Toll-6 and Toll-8, are adjacent in both the *D. melanogaster* and *A. mellifera* genomes.

### Immune effector diversity

Among the immune effectors, the total of six honey bee antimicrobial peptides contrasts with the 20 and nine found in *Drosophila* and *Anopheles*, respectively (Table 1). Two of these (abaecin, and apidaecin) are in the class of proline-rich antimicrobial peptides, two are conventional defensins and two (apisimin and hymenoptaecin) are distinct from all other recognized antimicrobial peptides. Genomic analysis reveals that the gene encoding apidaecin consists of a conserved N-terminus followed by several exons, each of which encodes a complete 28-amino acid peptide. Peptide and cDNA evidence for this gene was used to predict a mechanism for ratcheting up expression of apidaecin in response to bacterial challenge (Casteels-Josson *et al*., 1993). The genomic structure of apidaecin raises the possibility of a mechanism for generating specific responses to pathogens by splice variation. As each exon is a functional and distinct antimicrobial peptide, it is conceivable that splice variation at this locus can further refine this gene as an immune effector. Apidaecin exons differ greatly across individual honey bees in both number and in their encoded amino acid sequences. The two bee haplotypes sequenced in this project differed in sequence and exon number and also differed from three previously described apidaecin cDNAs (Casteels *et al*., 1993). Collectively, the two haplotypes sequenced here, and the three described by Casteels *et al.* encode a range of 4–11 secreted peptides each. While some peptides are shared between the various haplotypes for this gene, there is a surprisingly high level of sequence variation, such that the 35 peptides expressed by these five haplotypes reflect 23 different amino acid variants.

With the exception of defensin-2 (identified from genomic sequences generated during this project; Klaudiny *et al*., 2005) all of the honey bee antimicrobial peptides were first characterized by protein sequencing (Casteels-Josson *et al*., 1994), a fact that belies the difficulty in discovering such genes by sequence similarity across the millions of years separating insect species. Still, there is no evidence for close paralogues for any of the honey bee antimicrobial peptides, in contrast to such patterns in other insects (e.g. the gene-rich cecropin family). Five of the six bee antimicrobial peptides are up-regulated across diverse immune challenges (Fig. 3; Evans, 2004). Honey bees possess only one prophenoloxidase (proPO) gene, versus three and nine in *Drosophila* and *Anopheles*, respectively. Like most proPO, the honey bee proPO lacks a signal peptide and has the consensus sequence of NRFG around the activation site. The gene encoding proPO is expressed more strongly in older honey bee larvae and pupae (Lourenço *et al*., 2005). This gene was not up-regulated in our challenge experiments, but a gene identified as a proPO activator was up-regulated during natural infection (Fig. 3). There are only three lysozymes in the honey bee genome, two c-(chicken) type and one i-(invertebrate) type. One of the c-class lysozymes is up-regulated by challenged honey bees (Fig. 3).

There were fewer thiolester-containing proteins (TEPs) in the bee genome (four) than expected based on flies (Table 1). The *Anopheles* genome encodes 15 TEPs, most of them originating from species-specific expansion (Christophides *et al*., 2004) versus six members of this gene family in *Drosophila* (Agaisse & Perrimon, 2004). TEPs are induced after septic injury and promote phagocytosis in mosquitoes (Blandin & Levashina, 2004; Moita *et al*., 2005). They also play a central role in vertebrate innate immunity as the complement factors. In *Anopheles*, TEP1 was found to promote phagocytosis of Gram-negative bacteria and is also a major player in the host response to plasmodium infection. Members of this group are implicated as both recognition proteins and effectors (opsonins) in insects (Levashina *et al*., 2001; Moita *et al*., 2005; Stroschein-Stevenson *et al*., 2006).

## Discussion

As social animals, honey bees are at considerable risk from parasites and pathogens. Specifically, increased genetic relatedness and the high population densities that typify honey bee societies can strongly favour pathogen spread and epizootic outbreak. However, such social costs to honey bee immunity might be offset by social defences including mating strategy (e.g. multiple mating by queens: Tarpy, 2003), mutual grooming, and the maintenance of a sheltered environment for colony members. Given their well-studied natural pathogens, immune pathway models from the current annotated draft genome and unique genetic traits, honey bees can join with several fly and moth species as important systems with which to understand the genetic causes of immunity and disease. They also join humans as organisms for which there is great interest in understanding the social drivers of disease, and in using this information to improve host survival.

Our analyses indicate that the basic set of molecules defining the insect host-defence system is present in honey bees, including intact pathways for the key processes implicated in immunity and development. Single orthologues can be assigned for many pathway members, while others show several potential bee genes for which further work is needed to confirm roles. Interestingly however, whereas in *Drosophila* and *Anopheles* the host appears to have diversified its molecular arsenal through species-biased gene duplication (Table 1), we have not found examples of such gene expansions in honey bees. Thus, despite having wide-ranging parasites and pathogens, and tremendous losses to these pests at least in domesticated settings (Morse & Flottum, 1997), bees appear to have relatively diminished capacities to respond to and defend against pathogens.

Our first hypothesis to help explain this observation, that bee gene families are systematically smaller than in other insects, is not supported because there is no evidence for a systematic downward bias in paralogue counts across the bee genome at the level seen for immune-gene families (Honey Bee Genome Sequencing Consortium, 2006). Hypothesis two, that bee genes were simply missed due to sequence divergence, should apply particularly for immune genes that are short and subject only to limited sequence constraints, such as those encoding antimicrobial peptides. Indeed, few such peptides have been identified from bees (*n* = 6) and the means with which these few were discovered (directly at the protein level in all but one case) support the idea that sequence-level searches might have missed additional family members. Nevertheless, members of the remaining gene-poor families do not comprise especially short genes, nor genes divergent enough to be missed completely by comparisons at the level of insect orders. In fact, at least one significant bee:*Drosophila*:*Anopheles* orthologue is present in each of the remaining discussed families, indicating sufficient sequence-level conservation for identification of bee counterparts. Our third hypothesis, that bees simply have an undescribed mechanism for broadening their immune efficacy, is not testable at this point but would be very surprising given similarities in immune actors across the diverse insect orders studied to date.

Two of our initial hypotheses, that bees face a less diverse set of successful parasites and pathogens, and that societal defences by bees lessen pathogen pressures, therefore seem best supported by the data in hand and can now be compared. On one level, bee pathogens are diverse, ranging from Gram-positive and Gram-negative bacteria to fungi, RNA viruses, microsporidia and amoebae (Morse & Flottum, 1997). Bees are also parasitized by mites and other arthropods, raising risks of both pathogen infection (Kanbar & Engels, 2003; Chen *et al*., 2004) and lower abilities to combat disease (Gregory *et al*., 2005; Yang & Cox-Foster, 2005). Still, despite having pests that range over several kingdoms, common disease agents in bees are in fact restricted to several pathogens, two of which (the bacterium *Paenibacillus larvae* and the fungus *Ascosphaera apis*) are predominant. While functional data on the specificity of responses toward these and other pathogens are not yet available, gene-expression changes after challenge do not appear to be especially precise with respect to honey bee pathogens vs. exotics (e.g. *Escherichia coli*) or stress generally (Fig. 3). Thus, there is no compelling evidence at this point that the bee immune response is channelled just toward a small set of ‘true’ pathogens.

With respect to the final hypothesis, bees, like many social insects, are relentlessly hygienic, removing alien organisms from their nests, and secreting antimicrobial substances that can reduce the viability and growth of pathogens in the colonies. Bees also raise their young in individual cells using, as a food source, substances with strongly antimicrobial properties (e.g. royal jelly; Albert & Klaudiny, 2004). A testament to this hygiene is the fact that, even when facing severe colony-level infections by bacterial pathogens such as *Paenibacillus larvae* (for which < 10 spores are normally fatal to young larvae (Brodsgaard *et al*., 1998), the vast majority of larvae show no signs of exposure (Evans & Pettis, 2005). ‘Social’ barriers might also reduce exposure to minor, opportunistic, pathogens or saprophytes that have been proposed as generalized targets of insect immune defences (Hultmark, 2003). While bees do carry an assemblage of microbes, and bacteria in particular (Gilliam, 1997), exposure to these microbes is arguably lower than in free-living *Drosophila* (decaying plant material as larvae and adults) or *Anopheles* (septic aqueous environments as larvae). Further, bacteria found in bee colonies have only rarely been associated with disease pathologies, despite extensive study. In fact, some resident bacteria in colonies appear to add to external defences through their inhibition of bee pathogens (Evans & Armstrong, 2006).

Future genomic work can help reveal whether other species of highly social insects, including ants, wasps, bees and termites, also appear to have more simplistic innate defence systems. More generally, social and solitary insects with more ‘exposed’ life histories are predicted to have a greater number and higher functional diversity of immune-pathway genes and end products, when compared to sister taxa that are more sheltered. Data on parasite and pathogen abundance across social (e.g. Boomsma *et al*., 2005) and solitary insects could be used as a surrogate for disease loads in different taxa, although field and epidemiological data are most needed to assess the relative fitness impacts of disease and the efficacies of different lines of defence.

Through this analysis, we present the first plausible models for immune pathways in a social insect, the honey bee. We show nearly complete conservation of candidate genes for these pathways yet show that bees have consistently undercut numbers of genes that embellish these pathways in other insects. Genome-wide expression studies newly available for bees, and the proven success of gene knockdown techniques such as RNA inactivation (Lourenço *et al*., 2005), allow for more refinement of the roles played by pathway members as well as the discovery completely novel players in honey bee immunity. The latter discoveries, combined with analyses across more species of social and solitary bees, will help determine whether the observations described here are unique to the highly social honey bees. Longstanding agricultural interest has helped generate a wealth of data on honey bee pathologies (Morse & Flottum, 1997), and it is now possible to connect these data with immune traits that help limit pathogen efficacy. Through these connections, honey bees will provide a valuable and tractable model for disease transmission, immunity, ‘socialized medicine’ and pathology.

## Experimental procedures

### Bioinformatic screening of the honey bee genome

Immune-gene candidates from other insects were used in several ways to query the honey bee genome, primarily using the Blast family of search functions (http://www.ncbi.nlm.nih.gov). Most searches were initiated by BlastP queries against the consensus protein list (GLEAN3, derived from HBGP assembly 2.0) using BlastP and algorithms (BLOSUM and PAM variants) appropriate to gene size and structure. Honey bee orthologues were also identified by searching honey bee genome assemblies 2.0 and 3.0 directly using TBLASTN and either local databases or the BeeBase Blast server (http://racerx00.tamu.edu/blast/blast.html). Searches for missing genes were also carried out a on smaller coverage set of honey bee contigs that were too short to be included in the assembly, as well as the unassembled reads from the project (http://www.hgsc.bcm.tmc.edu/projects/honeybee/).

Given honey bee candidates, searches were repeated in the hope of identifying paralogues missed by interspecific comparisons. PSI-Blast was used to identify honey bee genes on the basis of conserved domains, followed by RPS-Blast to confirm the significance of these domain matches. Putative matches were aligned and, in the case of serine proteases, scavenger receptors and C-type lectins, screened for additional motifs using pfam categories (http://sanger.ac.uk/software/pfam). Tentative matches were aligned and checked for gene-prediction errors (in the case of genes from the official protein list) as part of the annotation of immune candidate genes for the Honey Bee Genome Project (Honey Bee Genome Sequencing Consortium, 2006). All protein matches were ported to Apis mellifera assembly 3.0 using the alignment program BLAT (Jim Kent, University California, Santa Cruz) to establish scaffold locations (Supplementary Material Table S1).

### Phylogenetic analyses

Best-match honey bee sequences were then aligned with counterparts from *D. melanogaster* and *A. gambiae*, along with other insects where sufficient genome-level data were available. Amino acid sequence alignments were carried out using GONNET series weight matrices, with the program CLUSTAL_X (Chenna *et al*., 2003). Alignments were used to propose phylogenetic relationships using maximum-parsimony and neighbour-joining algorithms, with the programs paup* (Sinauer, Sunderland, MA) or phylip (http://evolution.genetics.washington.edu/phylip.html). The PGRP tree is based on the conserved domain region only. Other alignments were edited manually to reduce or remove ambiguous regions. For scavenger receptor class B proteins, human (NP_005497) and mouse (NP_031669 CD36) proteins were added to alignments. Honey bee scavenger receptor AmelSCRB8 was not included because its relatively short predicted length (apparently the result of an intercontig gap) precluded an unambiguous alignment. All alignments are available on request.

### Gene-expression analyses

Two experiments were carried out to screen for immune-related transcript changes. In the first, adult worker bees from a single local *A. mellifera ligustica* colony were removed, then injected abdominally with either dilute phosphate-buffered saline or saline solution containing 103 live cells of *E. coli* or 103 vegetative spores of the honey bee bacterial pathogen *Paenibacillus larvae*. These bees, along with uninjected controls, were maintained for 24 h at high humidity and 34 °C and then were immediately frozen at −70 °C prior to RNA extraction. To assess immune responses following natural infection, eight 1st-instar larval bees from the same stock were given per os challenges of *P. larvae* in their food [(50 spores/µl as described in Evans (2004)], then maintained 24 h at 34 °C and high humidity. Parallel control larvae were given the same food without bacterial spores. All samples were frozen at −80 °C following incubation.

RNA was extracted from whole abdomens of the adult bees using a standard TRIzol (Invitrogen, Carlsbad, CA, USA) procedure while RNA was extracted from individual larvae using the RNAqueous kit (Ambion, Austin, TX). RNAs were pooled by sample duration for the eight larvae challenged with the bacterial pathogen *P. larvae*, and the eight controls prior to cDNA synthesis, giving six RNA pools. DNA was removed from all extracts, then first-strand cDNA was synthesized as described by Evans (2004). Transcript abundances for these cDNAs were assayed by quantitative real-time PCR with an Icycler real-time PCR machine (Bio-Rad, Hercules, CA, USA). Primer pairs were designed to amplify 120–300 bp sections of 39 honey bee immune-related genes derived from Supplementary Material Table S1 and ribosomal protein S5 as a control gene (primers in Supplementary Material Table S2). Primer sequences were modified, where necessary, to run in duplicate on 96-well plates using a fixed thermal protocol consisting of 5 min at 95 °C, then 40 cycles of a four-step protocol consisting of 94 °C for 20 s, 60 °C for 30 s, 72 °C for 1 min, and 78 °C for 20 s was used (Evans, 2006). Reactions were carried out on 0.5–2 µg cDNA along with 1 U Taq, the provided PCR buffer (Roche Applied Sciences, Indianapolis, IN, USA), 1 mm dNTP mix, 2 mm added MgCl_2_, 0.2 µm each primer, 1× concentration SYBR-Green I dye (Applied Biosystems, Foster City, CA, USA), and 10 nm fluorescein in a 25 µl reaction volume. Amplification was followed by a melt-curve dissociation program in order to confirm expected product size. Thresholds were calculated individually for each target gene on the 96-well plate. For adult bee samples, data were pooled for the three replicates in each single-bee injection treatment (or controls). Results were screened for the appropriate dissociation (melt-curve) values, and by 1% agarose gels, in order to ensure against primer artefacts and the presence of DNA contamination (which would have been evident for numerous primers spanning two exons). Immune-gene transcripts were normalized relative to expression levels for the gene encoding ribosomal protein S5, a gene with consistent expression across honey bee life stages and disease status (Evans & Wheeler, 2000; Evans, 2004). For display purposes, transcript abundance values (CTcontrol–CTtarget) for each gene were median-normalized across each panel of genes and clustered by average linkage clustering (using Cluster 3.0, M. Eisen, http://www.rana.lbl.gov/EisenSoftware.htm) and presented as relative grey-scale values (using Treeview, M. Eisen).

## Supplementary Material

Table S1 Immune-related genes annotated during the Honey Bee Genome Project.

Table S2 Locus (common) names, gene category (immune, development, control), primers and accession numbers for genes tested via quantitive RT-PCR for disease-induced transcriptional changes.

This material is available as part of the online article from http://www.blackwell-synergy.com

## References

[b1] Agaisse H, Perrimon N (2004). The roles of JAK/STAT signaling in Drosophila immune responses. Immunol Rev.

[b2] Agaisse H, Petersen U-M, Boutros M, Mathey-Prevot B, Perrimon N (2003). Signaling role of hemocytes in Drosophila JAK/STAT-dependent response to septic injury. Dev Cell.

[b3] Albert S, Klaudiny J (2004). The MRJP/YELLOW protein family of Apis mellifera: Identification of new members in the EST library. J Insect Physiol.

[b4] Aronstein K, Saldivar E (2005). Characterization of a honey bee Toll related receptor gene Am18w and its potential involvement in antimicrobial immune defense. Apidologie.

[b5] Baeg GH, Zhou R, Perrimon N (2005). Genome-wide RNAi analysis of JAK/STAT signaling components in Drosophila. Genes Dev.

[b6] Beutler B (2004). Innate immunity: an overview. Biochem Mol Immunol.

[b7] Bilak H, Tauszig-Delamasure S, Imler JL (2003). Toll and Toll-like receptors in Drosophila. Biochem Soc Trans.

[b8] Blandin S, Levashina EA (2004). Thioester-containing proteins and insect immunity. Mol Immunol.

[b9] Boomsma JJ, Schmid-Hempel P, Hughes WO.H, Fellowes MHG, Rolff J (2005). Life histories and parasite pressure across the major groups of social insects. Insect Evolutionary Ecology.

[b10] Boutros M, Agaisse H, Perrimon N (2002). Sequential activation of signaling pathways during innate immune responses in Drosophila. Dev Cell.

[b11] Brodsgaard CJ, Ritter W, Hansen H (1998). Response of in vitro reared honey bee larvae to various doses of Paenibacillus larvae larvae spores. Apidologie.

[b12] Brown CR, Brown MB (2004). Empirical measurement of parasite transmission between groups in a colonial bird. Ecology.

[b13] Burgett DM, Morse RA, Flottum K (1997). Antibiotic systems in honey, nectar, and pollen. Honey Bee Pest, Predators, and Diseases.

[b14] Casteels-Josson K, Capaci T, Casteels P, Tempst P (1993). Apidaecin multipeptide precursor structure: a putative mechanism for amplification of the insect antibacterial response. Embo J.

[b15] Casteels-Josson K, Zhang W, Capaci T, Casteels P, Tempst P (1994). Acute transcriptional response of the honeybee peptide-antibiotics gene repertoire and required post-translational conversion of the precursor structures. J Biol Chem.

[b16] Chang CI, Chelliah Y, Borek D, Mengin-Lecreulx D, Deisenhofer J (2006). Structure of tracheal cytotoxin in complex with a heterodimeric pattern-recognition receptor. Science.

[b17] Chen Y, Pettis JS, Evans JD, Feldlaufer MF, Kramer M (2004). Transmission of Kashmir bee virus by the ectoparasitic mite Varroa destructor. Apidologie.

[b18] Chenna R, Sugawara H, Koike T, Lopez R, Gibson TJ, Higgins DG, Thompson JD (2003). Multiple sequence alignment with the Clustal series of programs. Nucleic Acids Res.

[b19] Christe P, Oppliger A, Bancalà F, Castella G, Chapuisat M (2003). Evidence for collective medication in ants. Ecol Lett.

[b20] Christophides GK, Zdobnov E, Barillas-Mury C, Birney E, Blandin S, Blass C, Brey PT, Collins FH, Danielli A, Dimopoulos G, Hetru C, Hoa NT, Hoffmann JA, Kanzok SM, Letunic I, Levashina EA, Loukeris TG, Lycett G, Meister S, Michel K, Moita LF, Müller H-M, Osta MA, Paskewitz SM, Reichhart J-M, Rzhetsky A, Troxler L, Vernick KD, Vlachou D, Volz J, Von Mering C, Xu J, Zheng L, Bork P, Kafatos FC (2002). Immunity-related genes and gene families in Anopheles gambiae. Science.

[b21] Christophides GK, Vlachou D, Kafatos FC (2004). Comparative and functional genomics of the innate immune system in the malaria vector Anopheles gambiae. Immunol Rev.

[b22] Dostert C, Jouanguy E, Irving P, Troxler L, Galiana-Arnoux D, Hetru C, Hoffmann JA, Imler JL (2005). The Jak-STAT signaling pathway is required but not sufficient for the antiviral response of Drosophila. Nature Immunol.

[b23] Ekengren S, Hultmark D (2001). A family of Turandot-related genes in the humoral stress response of Drosophila. Biochem Biophys Res Comms.

[b24] Ekengren S, Tryselius Y, Dushay MS, Liu G, Steiner H, Hultmark D (2001). A humoral stress response in Drosophila. Curr Biol.

[b25] Evans JD (2004). Transcriptional immune responses by honey bee larvae during invasion by the bacterial pathogen, Paenibacillus larvae. J Invertebr Pathol.

[b26] Evans JD (2006). BeePath: An ordered quantitative-PCR array for exploring honey bee immunity and disease.. J Invertebr Pathol.

[b27] Evans JD, Armstrong TN (2006). Antagonistic interactions between honey bee bacterial symbionts and implications for disease. BMC Ecol.

[b28] Evans JD, Pettis JS (2005). Colony-level effects of immune responsiveness in honey bees, Apis mellifera. Evolution.

[b29] Evans JD, Wheeler DE (2000). Expression profiles during honeybee caste determination. Genome Biol.

[b30] Gilliam M (1997). Identification and roles of non-pathogenic microflora associated with honey bees. FEMS Microbiol Lett.

[b31] Graveley BR, Kaur A, Rowen L, Gunning D, Lawrence Zipursky S, Clemens JC (2004). The organization and evolution of the Dipteran and Hymenopteran Down syndrome cell adhesion molecule (Dscam) genes. RNA.

[b32] Gregory PG, Evans JD, Rinderer T, De Guzman L (2005). Conditional immune-gene suppression of honeybees parasitized by Varroa mites. J Insect Sci.

[b33] Hoffmann JA (2003). The immune response of Drosophila. Nature.

[b34] Honey Bee Genome Sequencing Consortium (2006). Insights into social insects from the genome of the honeybee *Apis mellifera*. Nature.

[b35] Hultmark D (2003). Drosophila immunity: Paths and patterns. Curr Opin Immunol.

[b36] Irving P, Ubeda JM, Doucet D, Troxler L, Lagueux M, Zachary D, Hoffmann JA, Hetru C, Meister M (2005). New insights into Drosophila larval haemocyte functions through genome-wide analysis. Cell Microbiol.

[b37] Jiang H, Kanost MR (2000). The clip-domain family of serine proteinases in arthropods. Insect Biochem Mol Biol.

[b38] Jiang H, Wang Y, Guo X, Zou Z, Gu Y, Kanost MR, Scholz F, Trenczek TE (2005). Molecular identification of a bevy of serine proteinases in Manduca sexta hemolymph. Insect Biochem Mol Biol.

[b39] Kambris Z, Hoffmann JA, Imler J-L, Capovilla M (2002). Tissue and stage-specific expression of the Tolls in Drosophila embryos. Gene Expr Patterns.

[b40] Kanbar G, Engels W (2003). Ultrastructure and bacterial infection of wounds in honey bee (Apis mellifera) pupae punctured by Varroa mites. Parasitol Res.

[b41] Kanost MR, Clarke T, Gilbert L I, Gilbert K I, Gill S (2005). Proteases. Comprehensive Molecular Insect Science.

[b42] Kanzok SM, Hoa NT, Bonizzoni M, Luna C, Huang Y, Malacrida AR, Zheng L (2004). Origin of Toll-like receptor-mediated innate immunity. J Mol Evol.

[b43] Klaudiny J, Bachanová KS, Simuth J, Albert S, Kopernický J (2005). Two structurally different defensin genes, one of them encoding a novel defensin isoform, are expressed in honeybee Apis mellifera. Insect Biochem Mol Biol.

[b44] Kocks C, Cho JH, Nehme N, Ulvila J, Pearson AM, Meister M, Strom C, Conto SL, Hetru C, Stuart LM, Stehle T, Hoffmann JA, Reichhart JM, Ferrandon D, Ramet M, Ezekowitz RA (2005). Eater, a transmembrane protein mediating phagocytosis of bacterial pathogens in Drosophila. Cell.

[b45] Lagueux M, Perrodou E, Levashina EA, Capovilla M, Hoffmann JA (2000). Constitutive expression of a complement-like protein in Toll and JAK gain-of-function mutants of Drosophila. Proc Natl Acad Sci USA.

[b46] Lazzaro BP (2005). Elevated polymorphism and divergence in the class C scavenger receptors of Drosophila melanogaster and D. simulans. Genetics.

[b47] Lemaitre B, Nicolas E, Michaut L, Reichhart J, Hoffmann J (1996). The dorsoventral regulatory gene cassette spätzle/Toll/cactus controls the potent antifungal response in Drosophila adults. Cell.

[b48] Levashina EA, Moita LF, Blandin S, Vriend G, Lagueux M, Kafatos FC (2001). Conserved role of a complement-like protein in phagocytosis revealed by dsRNA knockout in cultured cells of the mosquito, Anopheles gambiae. Cell.

[b49] Lourenço AP, Zufelato MS, Gentile Bitondi MM, Paulino Simões ZL (2005). Molecular characterization of a cDNA encoding prophenoloxidase and its expression in Apis mellifera. Insect Biochem Mol Biol.

[b50] Masuda N, Konno N, Aihara K (2004). Transmission of severe acute respiratory syndrome in dynamical small-world networks. Phys Rev E.

[b51] Mellroth P, Karlsson J, Håkansson J, Schultz N, Steiner H, Goldman WE (2005). Ligand-induced dimerization of Drosophila peptidoglycan recognition proteins in vitro. Proc Natl Acad Sci USA.

[b52] Moita LF, Wang-Sattler R, Michel K, Zimmermann T, Blandin S, Levashina EA, Kafatos FC (2005). Immunity.

[b53] Morse RA, Flottum K (1997). Honey Bee Pests Predators and Diseases. Honey Bee Pests Predators and Diseases.

[b54] Muller P, Kuttenkeuler D, Gesellchen V, Zeidler MP, Boutros M (2005). Identification of JAK/STAT signalling components by genome-wide RNA interference. Nature.

[b55] Ooi JY, Yagi Y, Hu X, Ip YT (2002). The Drosophila Toll-9 activates a constitutive antimicrobial defense. EMBO Rep.

[b56] Parker JS, Mizuguchi K, Gay NJ (2001). A family of proteins related to Spatzle, the toll receptor ligand, are encoded in the Drosophila genome. Proteins.

[b57] Reichhart JM (2005). Tip of another iceberg: Drosophila serpins. Trends Cell Biol.

[b58] Ross J, Jiang H, Wang Y, Kanost MR (2003). Serine proteases and their homologs in the Drosophila melanogaster genome: An initial analysis of sequence conservation and phylogenetic relationships. Gene.

[b59] Rothenbuhler WC (1964). Resistance to American foulbrood in honey bees: I. Differential survival of larvae of different genetic lines. Am Zool.

[b60] Royet J, Reichhart JM, Hoffmann JA (2005). Sensing and signaling during infection in Drosophila. Curr Opin Immunol.

[b61] Sadd BM, Kleinlogel Y, Schmid-Hempel R, Schmid-Hempel P (2005). Trans-generational immune priming in a social insect. Biol Lett.

[b62] Schmid-Hempel (1998). Parasites in Social Insects.

[b63] Schmid-Hempel P (2005). Evolutionary ecology of insect immune defenses. Annu Rev Entomol.

[b64] Shin SW, Kokoza V, Bian G, Cheon HM, Kim YJ, Raikhel AS (2005). REL1, a homologue of Drosophila dorsal, regulates toll antifungal immune pathway in the female mosquito Aedes aegypti. J Biol Chem.

[b65] Spivak M, Reuter GS (2001). Resistance to American foulbrood disease by honey bee colonies Apis mellifera bred for hygienic behavior. Apidologie.

[b66] Starks PT, Blackie CA, Thomas D, Seeley PT (2000). Fever in honeybee colonies. Naturwissenschaften.

[b67] Steiner H (2004). Peptidoglycan recognition proteins: On and off switches for innate immunity. Immunol Rev.

[b68] Stenbak CR, Ryu JH, Leulier F, Pili-Floury S, Parquet C, Herve M, Chaput C, Boneca IG, Lee WJ, Lemaitre B, Mengin-Lecreulx D (2004). Peptidoglycan molecular requirements allowing detection by the Drosophila immune deficiency pathway. J Immunol.

[b69] Stroschein-Stevenson SL, Foley E, Farrell PH, Johnson AD (2006). Identification of Drosophila Gene Products Required for Phagocytosis of Candida albicans. PLoS Biol.

[b70] Stuart LM, Ezekowitz RAB (2005). Phagocytosis: Elegant complexity. Immunity.

[b71] Tarpy DR (2003). Genetic diversity within honeybee colonies prevents severe infections and promotes colony growth. Proc Roy Soc Lond B – Biol Sci.

[b72] Tauszig S, Jouanguy E, Hoffmann JA, Imler J-L (2000). Toll-related receptors and the control of antimicrobial peptide expression in Drosophila. Proc Natl Acad Sci USA.

[b73] Traniello JFA, Rosengaus RB, Savoie K (2002). The development of immunity in a social insect: Evidence for the group facilitation of disease resistance. Proc Natl Acad Sci USA.

[b74] Watson FL, Puttmann-Holgado R, Thomas F, Lamar DL, Hughes M, Kondo M, Rebel VI, Schmucker D (2005). Extensive diversity of Ig-superfamily proteins in the immune system of insects. Science.

[b75] Werner T, Borge-Renberg K, Hultmark D, Mellroth P, Steiner H (2003). Functional diversity of the Drosophila PGRP-LC gene cluster in the response to lipopolysaccharide and peptidoglycan. J Biol Chem.

[b76] Wilson E (1971). The Insect Societies.

[b77] Wojda I, Kowalski P, Jakubowicz T (2004). JNK MAP kinase is involved in the humoral immune response of the greater wax moth larvae Galleria mellonella. Arch Insect Biochem Physiol.

[b78] Yang X, Cox-Foster DL (2005). Impact of an ectoparasite on the immunity and pathology of an invertebrate: Evidence for host immunosuppression and viral amplification. Proc Natl Acad Sci USA.

[b79] Zou Z, Lopez DL, Kanost MR, Evans JD, Jiang H (2006). Comparative analysis of serine protease-related genes in the honey bee genome: possible involvement in embryonic development and innate immunity. Insect Mol Biol.

