# Supplementary material for: Immune pathways and defence mechanisms in honey bees Apis mellifera
Source: Insect Mol Biol. 2006 Oct 1;15(5):645–56. doi: 10.1111/j.1365-2583.2006.00682.x (PMC1847501; doi:10.1111/j.1365-2583.2006.00682.x)
Supplement: Table S1 — Immune-related genes annotated during the Honey Bee Genome Project. [file imb0015-0645-ts1.doc]

Table S1. Immune-related genes annotated during the Honey Bee Genome Project

| Gene Name | Official Name | Gene Family/Pathway | ScaffoldAssembly4.0 | Orient | Start |
| --- | --- | --- | --- | --- | --- |
| abaecin | GB18323 | AMP | Group10.45-236395 | + | 84109 |
| Apidaecin | GB17782 | AMP | Group16.12+3740 | - | 2551343 |
| apisimin | GB19468 | AMP | Group6.44+518221 | + | 518221 |
| Defensin-1 | GB19392 | AMP | Group6.29+5011 | + | 5011 |
| Defensin-2 | GB10036 | AMP | Group16.16-7803 | - | 7152 |
| Hymenoptaecin | GB17538 | AMP | GroupUn.6281+1080 | + | 1080 |
| Hemolectin | GB16711 | Cellular response | Group8.26+18648 | + | 18648 |
| Hemomucin | GB20003 | Cellular response | Group5.14-56385 | - | 54243 |
| CTL1 | GB11717 | C-lectin domain | Group1.71+34115 | + | 34115 |
| CTL10 | GB11810 | C-lectin domain | GroupUn.67+568 | + | 568 |
| CTL11 | GB19013 | C-lectin domain | GroupUn.5963-3596 | - | 2759 |
| CTL12 | GB13808 | C-lectin domain | GroupUn.7291+31018 | + | 31018 |
| CTL2 | GB14265 | C-lectin domain | Group11.35+329528 | - | 23352 |
| CTL3 | GB18049 | C-lectin domain | Group11.1-259293 | + | 173691 |
| CTL4 | GB20122 | C-lectin domain | Group15.27+216404 | - | 129170 |
| CTL5 | GB17330 | C-lectin domain | Group8.12-30614 | - | 28944 |
| CTL6 | GB11792 | C-lectin domain | Group11.13-91112 | - | 78781 |
| CTL7 | GB14975 | C-lectin domain | GroupUn.284+4413 | + | 8383 |
| CTL8 | GB14382 | C-lectin domain | GroupUn.1315+11955 | + | 11955 |
| CTL9 | GB15050 | C-lectin domain | GroupUn.48-3375 | - | 2841 |
| Eater-like | GB14645 | EGF Family | Group15.26-52845 | + | 81509 |
| laminin-EGF-like | GB14962 | EGF Family | GroupUn.894+10481 | + | 10481 |
| laminin-EGF-like | GB94141 | EGF Family | Group15.18+361 | + | 361 |
| Angiopoietin | GB17018 | Fibrinogen | Group6.48-31530 | - | 22948 |
| Scabrous | GB11902 | Fibrinogen | Group1.33+148272 | - | 21758 |
| Galectin-1 | GB10026 | Galectin | Group5.22+780555 | + | 780555 |
| Galectin-2 | GB18324 | Galectin | Group1.38+123281 | - | 196728 |
| B-gluc1 | GB19452 | GNBPs | Group9.18-266750 | - | 263319 |
| B-gluc2 | GB19961 | GNBPs | Group4.16+1040 | - | 282733 |
| Dscam | GB30209 | IG superfamily genes | #N/A | - | 511302 |
| IGFn3-1 | GB14642 | IG Superfamily Genes | Group7.31-114069 | - | 40718 |
| IGFn3-10 | GB11807 | IG Superfamily Genes | Group4.27-307431 | + | 357054 |
| IGFn3-11 | GB12490 | IG Superfamily Genes | Group4.27-279903 | + | 384582 |
| IGFn3-12 | GB14520 | IG Superfamily Genes | Group10.15-245492 | - | 216575 |
| IGFn3-13 | GB10469 | IG Superfamily Genes | Group2.31+51661 | - | 592236 |
| IGFn3-14 | GB12933 | IG Superfamily Genes | Group5.11-67381 | - | 5007 |
| IGFn3-15 | GB15344 | IG Superfamily Genes | Group14.24+1193898 | - | 193301 |
| IGFn3-16 | GB11918 | IG Superfamily Genes | GroupUn.1229+5973 | + | 5973 |
| IGFn3-2 | GB11358 | IG Superfamily Genes | Group2.43+163200 | - | 311872 |
| IGFn3-3 | GB15987 | IG Superfamily Genes | Group6.2+73678 | + | 73678 |
| IGFn3-4 | GB11115 | IG Superfamily Genes | Group11.26-653375 | + | 37395 |
| IGFn3-5 | GB13261 | IG Superfamily Genes | Group4.23-1035798 | + | 39373 |
| IGFn3-6 | GB10912 | IG Superfamily Genes | Group3.22+210960 | - | 70375 |
| IGFn3-7 | GB11846 | IG Superfamily Genes | Group5.21-439542 | - | 433887 |
| IGFn3-8 | GB14317 | IG Superfamily Genes | Group3.27+64435 | + | 64435 |
| IGFn3-9 | GB16060 | IG Superfamily Genes | Group5.12-1223818 | - | 902876 |
| DREDD-Casp8 | GB30331 | IMD | Group6.52-84424 | - | 82490 |
| dUbc13 | GB19498 | IMD | GroupUn.17-5953 | - | 5265 |
| FADD | GB30399 | IMD | #N/A | - | 24874 |
| IKKgamma-kenny | GB17106 | IMD | Group13.15-473766 | - | 471905 |
| IKK-ird5 | GB15273 | IMD | Group2.6+21834 | + | 21834 |
| Imd | GB18606 | IMD | Group5.33-306420 | - | 305470 |
| lap2 | GB11057 | IMD | GroupUn.51-18069 | - | 15708 |
| relish | GB13742 | IMD | GroupUn.4651+301 | + | 301 |
| Tab | GB18650 | IMD | Group14.24+158940 | - | 1288745 |
| Tak1 | GB14664 | IMD | GroupUn.4555-13055 | - | 3652 |
| Domeless | GB12159 | JakSTAT | Group1.63-8993 | - | 3396 |
| D-PIAS | GB18362 | JakSTAT | Group5.11-426433 | - | 424973 |
| Hopscotch | GB16422 | JakSTAT | GroupUn.427+14968 | + | 14968 |
| SOCS | GB18949 | JakSTAT | GroupUn.298-4696 | - | 3538 |
| STAT92E | GB18923 | JakSTAT | Group15.34-20005 | + | 612249 |
| hem | GB17167 | JNK | Group3.7+85073 | - | 313063 |
| Jra | GB12004 | JNK | Group9.24+617099 | - | 209961 |
| Kay | GB12212 | JNK | Group5.13+98779 | + | 98779 |
| puckered | GB19901 | JNK | Group5.22-486364 | - | 411453 |
| Lys-1 | GB10231 | Lysozyme | Group13.9-97154 | - | 96680 |
| Lys-2 | GB15106 | Lysozyme | Group13.9-95782 | - | 95350 |
| Lys-3 | GB19988 | Lysozyme | GroupUn.99-11282 | - | 8893 |
| MAPK(basket) | GB16401 | MAPK | GroupUn.391-7237 | - | 2362 |
| MAPKKK9 | GB13522 | MAPK | Group11.29-77007 | - | 71878 |
| RIP1 | GB11320 | MAPK | Group4.7+1194677 | - | 598558 |
| NFAT | GB15645 | NFAT | Group11.31+1428784 | - | 714657 |
| NimA | GB12883 | Phagocytosis | Group15.26-64961 |  |  |
| NimC1 | GB14645 | Phagocytosis | Group15.26-52845 |  |  |
| NimB | GB12454 | Phagocytosis | GroupUn.6715-6981 |  |  |
| NimC2 | GB13979 | Phagocytosis | Group15.26-43212 |  |  |
| Draper | GB14962 | Phagocytosis | GroupUn.894+10481 |  |  |
| PGRP-LC | GB17188b | PGRP | Group7.38+904 | - | 643002 |
| PGRP-S1 | GB15371 | PGRP | Group13.13+1049608 | + | 1049608 |
| PGRP-S2 | GB19301 | PGRP | Group13.13+1052270 | + | 1052270 |
| PGRP-S3 | GB17879 | PGRP | GroupUn.165-13763 | - | 11910 |
| PPO | GB18313 | PPO | Group14.17+466991 | - | 374159 |
| PPOAct | GB18767 | PPO | Group15.34-125434 | + | 506820 |
| Rac | GB11373 | RAC1 protein | GroupUn.6813+2597 | + | 2597 |
| Corin-like | GB19649 | Scav. Receptor A | Group10.31+720913 | - | 10809 |
| GRAAL/Tequila-like | GB12538 | Scav. Receptor A | Group4.13-955292 | + | 384206 |
| lox2-like | GB13360 | Scav. Receptor A | Group15.21+90110 | + | 90110 |
| AmSCR-B1 | GB10506 | Scav. Receptor B | Group12.28-17961 | - | 1983 |
| AmSCR-B10 | GB19683 | Scav. Receptor B | GroupUn.649-28318 | - | 20574 |
| AmSCR-B2 | GB11743 | Scav. Receptor B | Group16.19-274949 | + | 470208 |
| AmSCR-B3 | GB12378 | Scav. Receptor B | GroupUn.649+12364 | + | 12364 |
| AmSCR-B4 | GB12830 | Scav. Receptor B | Group4.15-610302 | + | 225747 |
| AmSCR-B5 | GB13813 | Scav. Receptor B | GroupUn.1485-12033 | - | 1199 |
| AmSCR-B6 | GB14314 | Scav. Receptor B | Group4.27-265911 | + | 398574 |
| AmSCR-B7 | GB15549 | Scav. Receptor B | Group10.28-44412 | - | 36069 |
| AmSCR-B8 | GB16388 | Scav. Receptor B | GroupUn.415+6741 | + | 6741 |
| AmSCR-B9 | GB19916 | Scav. Receptor B | Group4.23+238063 | - | 834562 |
| AmSCR-C | GB19925 | Scav. Receptor C | Group8.20-136428 | - | 129240 |
| cSP1 | GB16147 | serine proteases | Group5.33+88855 | + | 88855 |
| cSP10 | GB17927 | serine proteases | Group15.22-10919 | - | 4212 |
| cSP14 | GB14044 | serine proteases | Group1.73+60353 | + | 60353 |
| cSP2 | GB14247 | serine proteases | Group5.33+86102 | + | 86102 |
| cSP21 | GB16220 | serine proteases | Group4.23-654172 | + | 420502 |
| cSP25 | GB19719 | serine proteases | Group13.6-610822 | - | 604444 |
| cSP26 | GB18450 | serine proteases | Group14.23+141045 | - | 831702 |
| cSP3 | GB11698 | serine proteases | Group4.8-219586 | + | 1675 |
| cSP33 | GB14309 | serine proteases | Group13.6-589115 | - | 584409 |
| cSP6 | GB14077 | serine proteases | Group13.3+46205 | + | 46205 |
| cSP7 | GB17145 | serine proteases | Group5.12+1508751 | + | 1508751 |
| cSPH39 | GB14366 | serine proteases | Group8.7-22436 | - | 9240 |
| cSPH41 | GB10943 | serine proteases | Group15.36-180497 | + | 24890 |
| cSPH42 | GB11298 | serine proteases | Group8.7-4447 | - | 2238 |
| cSPH50 | GB14001 | serine proteases | Group9.25+316380 | - | 226386 |
| cSPH55 | GB15254 | serine proteases | Group15.40-12628 | - | 4860 |
| SP11 | GB14654 | serine proteases | Group9.6-55437 | + | 195215 |
| SP12 | GB19856 | serine proteases | Group5.11+435219 | + | 435219 |
| SP13 | GB15640 | serine proteases | Group15.29+700740 | - | 650848 |
| SP15 | GB18178 | serine proteases | GroupUn.2558+2992 | + | 2992 |
| SP16 | GB12253 | serine proteases | Group15.25+284187 | - | 111576 |
| SP17 | GB14603 | serine proteases | Group13.10-466592 | - | 462848 |
| SP18 | GB10222 | serine proteases | Group6.11-7066 | + | 366340 |
| SP20 | GB19590 | serine proteases | Group8.18+188638 | - | 34140 |
| SP22 | GB13791 | serine proteases | Group6.10-23905 | - | 19976 |
| SP23 | GB12538 | serine proteases | Group4.13-955292 | + | 384206 |
| SP24 | GB14233 | serine proteases | Group1.66-13438 | - | 5531 |
| SP27 | GB11588 | serine proteases | Group15.33-84700 | + | 80019 |
| SP28 | GB13489 | serine proteases | Group4.23-911028 | + | 163646 |
| SP29 | GB14644 | serine proteases | Group9.6-63787 | + | 186865 |
| SP30 | GB19649 | serine proteases | Group10.31+720913 | - | 10809 |
| SP31 | GB11297 | serine proteases | Group1.54-100507 | - | 99435 |
| SP32 | GB11511 | serine proteases | Group13.10-462195 | - | 461240 |
| SP34 | GB11552 | serine proteases | GroupUn.98-21401 | - | 17929 |
| SP35 | GB16021 | serine proteases | GroupUn.1487-6682 | - | 4045 |
| SP36 | GB19846 | serine proteases | Group2.20-368025 | + | 511201 |
| SP38 | GB16214 | serine proteases | Group10.3-188191 | - | 185748 |
| SP4 | GB10646 | serine proteases | Group9.6-50932 | + | 199720 |
| SP40 | GB13263 | serine proteases | Group6.20+237518 | - | 5390 |
| SP43 | GB18530 | serine proteases | GroupUn.5543+132 | + | 132 |
| SP44 | GB15453 | serine proteases | Group15.27-80044 | + | 281697 |
| SP45 | GB17654 | serine proteases | Group10.21-10526 | - | 1793 |
| SP46 | GB16367 | serine proteases | Group1.18-467370 | - | 464946 |
| SP47 | GB14774 | serine proteases | Group6.10+5554 | + | 5554 |
| SP48 | GB12379 | serine proteases | Group8.19-332251 | - | 330927 |
| SP49 | GB15317 | serine proteases | Group6.18-73424 | - | 69388 |
| SP5 | GB12300 | serine proteases | Group9.6+71652 | - | 175424 |
| SP8 | GB18767 | serine proteases | Group15.34-125434 | + | 506820 |
| SP9 | GB18732 | serine proteases | GroupUn.125+2608 | + | 2608 |
| SPH19 | GB17345 | serine proteases | Group14.24-896836 | + | 555392 |
| SPH37 | GB18944 | serine proteases | Group15.9-15522 | - | 13712 |
| SPH51 | GB13397 | serine proteases | Group9.6-44985 | + | 205667 |
| SPH52 | GB19292 | serine proteases | Group6.51+163487 | + | 163487 |
| SPH53 | GB15702 | serine proteases | Group14.8-325440 | - | 324274 |
| SPH54 | GB15980 | serine proteases | Group9.17+70833 | - | 219515 |
| SPH56 | GB13019 | serine proteases | Group13.4+305943 | + | 305943 |
| SPH57 | GB16038 | serine proteases | Group6.10-17290 | - | 7188 |
| TEP7 | GB12605 | TEP | Group7.34+18592 | + | 18592 |
| TEPA | GB18789 | TEP | GroupUn.1143-7602 | - | 1990 |
| TEPB | GB11563 | TEP | GroupUn.1310+5926 | + | 5926 |
| 18-w | GB15177 | Toll/TLR | Group2.16-122360 | - | 118247 |
| cact-1 | GB10655 | Toll/TLR | Group4.23+301244 | - | 769331 |
| cact-2 | GB13520 | Toll/TLR | Group4.22-686566 | + | 64838 |
| cact-3 | GB11883 | Toll/TLR | Group9.24+459489 | - | 366686 |
| cactin | GB13677 | Toll/TLR | Group5.12+871756 | + | 871756 |
| dorsal-1A | GB19066 | Toll/TLR | Group4.22+580515 | - | 164583 |
| Dorsal-1B | GB19537 | Toll/TLR | Group4.22+580515 | - | 168073 |
| Dorsal-2 | GB18032 | Toll/TLR | Group7.35+1084238 | - | 29073 |
| Mik2 | GB14720 | Toll/TLR | Group7.34-15780 | - | 13293 |
| MyD88 | GB12344 | Toll/TLR | Group3.16+58891 | + | 58891 |
| NEC LIKE | GB16472 | Toll/TLR | GroupUn.710-4201 | - | 2745 |
| NEC LIKE | GB17012 | Toll/TLR | GroupUn.329+31720 | + | 31720 |
| NEC LIKE | GB19582 | Toll/TLR | Group10.23+9104 | + | 9104 |
| pelle | GB16397 | Toll/TLR | GroupUn.1226-2636 | - | 714 |
| Pellino | GB16970 | Toll/TLR | Group11.34+44793 | + | 44793 |
| PSH LIKE | GB14044 | Toll/TLR | Group1.73+60353 | + | 60353 |
| PSH LIKE | GB15640 | Toll/TLR | Group15.29+700740 | - | 650848 |
| PSH LIKE | GB17927 | Toll/TLR | Group15.22-10919 | - | 4212 |
| PSH LIKE | GB18732 | Toll/TLR | GroupUn.125+2608 | + | 2608 |
| SPZ | GB13503 | Toll/TLR | Group12.27-494561 | - | 493628 |
| SPZ | GB15688 | Toll/TLR | Group10.33-40998 | - | 40166 |
| Toll (TLR) | GB18520 | Toll/TLR | Group4.14-11459 | - | 6803 |
| Toll-10 | GB16299 | Toll/TLR | Group10.29-251315 | + | 166594 |
| Toll-6 | GB17781 | Toll/TLR | Group10.30-350830 | + | 188295 |
| Toll-8/Trex | GB10640 | Toll/TLR | Group10.30-165959 | + | 373166 |
| Tollip | GB17961 | Toll/TLR | Group8.10-24434 | - | 22585 |
| TRAF2 | GB10539 | Toll/TLR | GroupUn.677+3072 | + | 3072 |
| tube | GB15684 | Toll/TLR | Group8.9-12233 | - | 11220 |
