# Supplementary material for: Immune pathways and defence mechanisms in honey bees Apis mellifera
Source: Insect Mol Biol. 2006 Oct 1;15(5):645–56. doi: 10.1111/j.1365-2583.2006.00682.x (PMC1847501; doi:10.1111/j.1365-2583.2006.00682.x)
Supplement: Table S2 — Locus (common) names, gene category (immune, development, control), primers and accession numbers for genes tested via quantitive RT-PCR for disease-induced transcriptional changes. [file imb0015-0645-ts2.doc]

Table S2. Locus (common) names, gene category (immune, development, control), primers and accession numbers for genes tested via quantitative RT-PCR for disease-induced transcriptional changes.

| Locus | Gene ID | Cat | Forward Primer | Reverse Primer |
| --- | --- | --- | --- | --- |
| abaecin | GB18323 | Imm | CAGCATTCGCATACGTACCA | GACCAGGAAACGTTGGAAAC |
| Am52C8 | GB10869 | Dev | GGACAATTGGATCTGCTCGT | GTGTTGCTTCCGCTTTTCAG |
| AmPPO | GB18313 | Imm | AGATGGCATGCATTTGTTGA | CCACGCTCGTCTTCTTTAGG |
| Apidaec | GB17782 | Imm | TAGTCGCGGTATTTGGGAAT | TTTCACGTGCTTCATATTCTTCA |
| apisimin | GB19468 | Imm | TGAGCAAAATCGTTGCTGTC | AACGACATCCACGTTCGATT |
| Bgluc1 | GB19492 | Imm | GGACAACCACCTTTTGAACG | AGGAGCTTCCTCTGCACTGA |
| basket | GB16401 | Imm | AGGAGAACGTGGACATTTGG | AATCCGATGGAAACAGAACG |
| cactus-1 | GB10655 | Imm | CACAAGATCTGGAGCAACGA | GCATTCTTGAAGGAGGAACG |
| cactus-2 | GB13520 | Imm | TTAGCAGGACAAACGGCTCT | CAGAAAGTGGTTCCGGTGTT |
| defensin2 | GB10036 | Imm | GCAACTACCGCCTTTACGTC | GGGTAACGTGCGACGTTTTA |
| defensin1 | GB19392 | Imm | TGCGCTGCTAACTGTCTCAG | AATGGCACTTAACCGAAACG |
| domeless | GB16422 | Imm | TTGTGCTCCTGAAAATGCTG | AACCTCCAAATCGCTCTGTG |
| dorsal-1 | GB19537 | Imm | AAATGGTTCGCTCGTAGCAC | TCCATGATATGAGTGATGGAAA |
| dorsal-2 | GB18032 | Imm | TCACCATCAACGCCTAACAA | AACTAACACCACGCGCTTCT |
| Dredd | GB17683 | Imm | GCGTCATAAAGAAAAAGGATCA | TTTCGGGTAATTGAGCAACG |
| Dscam | GB11871 | Imm | TTCAGTTCACAGCCGAGATG | ATCAGTGTCCCGCTAACCTG |
| EGFlikeA | GB14645 | Imm | CATTTGCCAACCTGTTTGT | ATCCATTGGTGCAATTTGG |
| hemipterous | GB17161 | Imm | CACCTGTTCAGGGTGGATCT | CCTTCGTGCAAAAGAAGGAG |
| hopscotch | GB12159 | Imm | ATTCATGGCATCGTGAACAA | CTGTGGTGGAGTTGTTGGTG |
| hymenopt | GB17538 | Imm | ctcttctgtgccgttgcata | GCGTCTCCTGTCATTCCATT |
| Imd | GB18606 | Imm | TGTTAACGACCGATGCAAAA | CATCGCTCTTTTCGGATGTT |
| Kenny | GB17106 | Imm | GCTGAACCAGAAAGCCACTT | TGCAAGTGATGATTGTTGGA |
| Lys-1 | GB10231 | Imm | GAACACACGGTTGGTCACTG | ATTTCCAACCATCGTTTTCG |
| Lys-2 | GB15106 | Imm | CCAAATTAACAGCGCCAAGT | GCAATTCTTCACCCAACCAT |
| Lys-3 | GB19988 | Imm | ATCTGTTTGCGGACCATTTC | TCGATGAATGCGAAGAAAATC |
| myd88 | GB12344 | Imm | TCACATCCAGATCCAACTGC | CAGCTGACGTTTGAGATTTTTG |
| perseph | GB17927 | Imm | CCGGTGAACTTGGAAAAGAT | CTTCGCCAGGAATAATGGAA |
| PGRP-S1 | GB17879 | Imm | TTTGAAAATTTCCTATGAAAGCA | TTTTTAATTGGTGGAGATGGAAA |
| PGRP-LC | GB17188 | Imm | TCCGTCAGCCGTAGTTTTTC | CGTTTGTGCAAATCGAACAT |
| PGRP-S2 | GB19301 | Imm | TAATTCATCATTCGGCGACA | TGTTTGTCCCATCCTCTTCC |
| PGRP-S3 | GB15371 | Imm | GAGGCTGGTACGACATTGGT | TTATAACCAGGTGCGTGTGC |
| PPOact | GB18767 | Imm | GTTTGGTCGACGGAAGAAAA | CCGTCGACTCGAAATCGTAT |
| relish | GB13742 | Imm | GCAGTGTTGAAGGAGCTGAA | CCAATTCTGAAAAGCGTCCA |
| RPL8 | GB17629 | Con | TGGATGTTCAACAGGGTTCATA | CTGGTGGTGGACGTATTGATAA |
| RPS5 | GB11132 | Con | AATTATTTGGTCGCTGGAATTG | TAACGTCCAGCAGAATGTGGTA |
| spaetzle | GB15688 | Imm | TGCACAAATTGTTTTTCCTGA | GTCGTCCATGAAATCGATCC |
| tab | GB18650 | Imm | GCTATCATGCAGCTGTTCCA | ACACTGGGTCAGCCAATTTC |
| Tak1 | GB14664 | Imm | ATGGATATGCTGCCAATGGT | TCGGATCGCATTCAACATAA |
| TEP7 | GB12605 | Imm | GAGCCTACAGCCTCGTTTTG | CGGTTTCACGATTACGTCCT |
| TEPA | GB18789 | Imm | CAAGAAGAAACGTGCGTGAA | ATCGGGCAGTAAGGACATTG |
| Toll | GB18520 | Imm | TAGAGTGGCGCATTGTCAAG | ATCGCAATTTGTCCCAAAAC |
